# Early Laparoscopic Cholecystectomy for Acute Gallstone Pancreatitis: Retrospective Review of Factors Influencing Timing of Surgery and Outcomes in a Single Institution

**DOI:** 10.7759/cureus.116334

**Published:** 2026-09-16

**Authors:** Leon Yeung, Amos Nepacina Liew, Mason Lee, Mariam Mohammad Rahim, Andrew D Gray

**Affiliations:** 1 General Surgery and Hepatobiliary Surgery, Monash Health, Melbourne, AUS

**Keywords:** early cholecystectomy, gallstone pancreatitis, index admission cholecystectomy, laparoscopic cholecystectomy (lc), optimal surgical timing

## Abstract

Introduction

Early laparoscopic cholecystectomy (ELC) is considered best practice in the treatment of acute gallstone pancreatitis. However, there is debate regarding the timing of surgery during the index admission. We reviewed some of the factors that can help determine the timing of early cholecystectomy, and its impact on patient outcomes.

Methods

Electronic medical records were retrospectively reviewed for all adult patients with acute gallstone pancreatitis who underwent laparoscopic cholecystectomy during the same admission over a two-year period at a tertiary centre. Patient outcomes and complication rates were compared based on whether they met our preoperative study criteria of being opioid-free, tolerating a solid diet, and having a serum C-reactive protein (CRP) level ≤100mg/L.

Results

A total of 199 patients were included in our study, of whom 83 (42%) met all three of our preoperative criteria, while the remaining 116 (58%) did not meet at least one of these criteria. The median time to surgery was four days (interquartile range (IQR) 3-6) and median length of stay was six days (IQR 4-8), which was similar between the two study groups. There was no significant difference found in rates of postoperative surgical site infection, haematoma, bile leak, and intra-abdominal collection. There were also similar rates of further interventions, including endoscopic retrograde cholangiopancreatography (ERCP) and interventional radiology. There was no postoperative venous thromboembolism or mortality reported in either study group.

Conclusion

This study highlights the relatively low complication rates of ELC for acute gallstone pancreatitis, as well as some of the difficulty in determining the appropriate time to proceed to ELC during the index admission. While patient factors (e.g. diet tolerance and pain level) and biochemical markers (e.g. serum CRP level) have been considered as indicators of readiness for surgery, using these has not necessarily demonstrated significant differences in patient outcomes or complication rates. It should therefore be considered whether ELC can be safely performed without waiting for all of these preoperative factors to be met, which would likely lead to a reduced hospital stay and, therefore, potentially reduced individual healthcare costs.

## Introduction

Acute pancreatitis is a common acute surgical condition, with an incidence range of 4.9-25 per 100000 population worldwide [1]. Approximately 40% of acute pancreatitis cases in Western countries are caused by gallstones, termed acute gallstone/biliary pancreatitis [2]. In Australia between 2006 and 2019 inclusive, there were 208,390 hospital admissions for acute pancreatitis, with the main underlying aetiologies being biliary (24.1% of acute pancreatitis hospital admissions), alcohol-related (20.3%), and idiopathic (51.6%) causes [3]. 

Management of acute gallstone pancreatitis (AGP) is usually guided by its severity based on the revised Atlanta classification [4]. Under this classification, mild pancreatitis is defined as having no organ failure and no local or systemic complications; moderate pancreatitis has transient organ failure that resolves within 48 hours, or has local or systemic complications without persistent organ failure, and severe pancreatitis demonstrates persistent organ failure greater than 48 hours.

In mild AGP, it is commonly accepted that early laparoscopic cholecystectomy (ELC) is the optimal definitive treatment, while in severe AGP, an interval or delayed laparoscopic cholecystectomy (DLC) may be preferred [5]. For the purposes of this study, ELC is defined as a laparoscopic cholecystectomy performed during the index admission for AGP, while DLC is defined as being performed in a subsequent admission after the index admission.

Despite the evidence suggesting that ELC should be a routine part of the definitive treatment of mild AGP, it is not necessarily commonplace in practice. In a retrospective cohort study, Blundell et al. found that in New South Wales, Australia, between 2008 and 2018, only 37.9% of mild AGP cases treated with cholecystectomy underwent ELC during their index admission, with the remainder receiving DLC [6]. Various population-based studies conducted in other countries, including the United Kingdom and the United States, showed that patients with mild AGP only received ELC in 10-60% of cases [7]. 

In addition, few studies have specifically investigated which clinical factors and variables can be used to determine the appropriate timing for ELC. Da Costa et al. conducted the randomised controlled PONCHO (Pancreatitis of biliary Origin: optimal timiNg of CHOlecystectomy) trial of 266 patients with mild AGP [8]. They included adult patients who were tolerating an oral diet, did not require opioid analgesics, and had a serum C-reactive protein (CRP) level ≤100mg/L preoperatively. The patients were then randomised to either ELC during the index admission (within three days) or DLC (25-30 days from randomisation). They found a significantly higher risk of readmission due to gallstone-related complications in the DLC group compared to the ELC group.

This study aims to review the timing for ELC within the index admission for patients with AGP, based on multiple factors including clinical condition, biochemical markers, and analgesia requirements. It also aims to compare the impact of surgery timing on postoperative outcomes and complications. Improved understanding of these preoperative factors can likely lead to earlier identification of patients who are ready for ELC during their index admission, which could further improve patient outcomes and possibly reduce individual healthcare system costs.

This article was previously presented as a meeting abstract at the 2026 Royal Australasian College of Surgeons’ Annual Scientific Congress in Perth, Western Australia, on May 1, 2026.

## Materials and methods

A retrospective analysis was performed on patients from Monash Health in Melbourne, Australia, from May 2022 to May 2024 (inclusive) using electronic medical records. This retrospective study was approved by the Monash Health Research Office (Research Support Services) (reference number: RES-24-0001-010Q). Due to the retrospective nature of the study and the use of de-identified patient data, the requirement for informed consent was waived.

Eligibility criteria

All adult patients diagnosed with AGP within the study period and who underwent laparoscopic cholecystectomy during their index hospital admission were included. All patients were diagnosed according to the revised Atlanta classification, requiring two out of the following three features: abdominal pain consistent with acute pancreatitis, serum lipase level at least three times greater than the upper limit of normal, and characteristic findings of acute pancreatitis on imaging [4]. Patients with pancreatitis from non-biliary aetiologies, or who had known chronic pancreatitis, were excluded from the study. In addition, patients who either declined or were deemed unsuitable for index admission surgery were also excluded. 

Data collection

The data collected included: patient demographics (age, sex), comorbidities, blood results (including white cell count and CRP levels) on admission and on the day of surgery, analgesia requirements on admission and prior to surgery (including whether the patients were opioid-free for at least 24 hours prior to surgery), diet regimen on admission and prior to surgery (including full ward diet, light ward diet, free fluid diet, clear fluid diet, sips only, or nil by mouth), presence of abdominal pain prior to proceeding to surgery, and time from admission to surgery.

The American Society of Anesthesiologists (ASA) Physical Status Classification System score [9] and Bedside Index of Severity in Acute Pancreatitis (BISAP) score [10] were also collected for each patient. The ASA score classifies patients from I to VI based on their overall health prior to surgery (I: normal health, II: mild systemic disease, III: severe systemic disease that is not incapacitating, IV: incapacitating disease that is a constant threat to life, V: moribund patient not expected to survive 24 hours without surgery, VI: brain-dead patient undergoing organ donation). The BISAP score predicts mortality risk from pancreatitis, with a score out of five based on receiving one point for each of the following factors during the first 24 hours: blood urea nitrogen (BUN) >25mg/dl, impaired mental status, systemic inflammatory response syndrome (SIRS), age >60 years, and presence of a pleural effusion. Mortality ranges from 0.1% for BISAP 0, 0.5% for BISAP 1, and up to 22.5% for BISAP 5.

Division into groups

Study participants were categorised into two groups based on whether they met preoperative criteria of being opioid-free for at least 24 hours prior to surgery, tolerating a solid diet prior to surgery, and having a serum CRP level ≤100 mg/L prior to surgery. The decision to proceed with ELC or DLC and the operative approach was at the discretion of the consultant surgeon.

Outcomes

The primary outcome measure was the rate of postoperative complications, as defined by the Clavien-Dindo classification of surgical complications [11], following ELC during an index admission for AGP. Secondary outcomes included duration of surgery, length of stay, further interventions required, and 30-day morbidity and mortality. 

Statistical analysis

Data analysis was performed using IBM SPSS Statistics for Windows, version 25 (IBM Corp., Armonk, New York, United States). Continuous non-parametric data were expressed as median values with ranges, and categorical data as numbers with percentages. Nominal or ordinal outcomes were compared using the Chi-square test. A p-value less than 0.05 was considered statistically significant. 

## Results

Patient selection and characteristics

A total of 199 patients were included with a diagnosis of AGP and an index admission laparoscopic cholecystectomy. Of these, 83 (41.7%) tolerated a solid diet, were opioid-free for 24 hours, and had a CRP level ≤100 mg/L prior to undergoing surgery. The remaining 116 (58.3%) patients did not fulfil at least one of these criteria. Of these, 49 (42.2%) were unable to tolerate a solid diet, 69 (59.5%) had an opioid analgesia requirement, and 43 (37%) had a CRP level ≥100 preoperatively. 

There was no significant difference in mean age between the patients who met preoperative criteria (55.2 years) and those with criteria not met (55.3 years) or in gender distribution, with the number of female participants in the first group being 56 (67.5%) and the number in the second group being 66 (56.9%) (p=0.131). The median ASA of the study cohort was 2, and the median BISAP score was 1. There was no significant difference between the groups in the proportion of patients with ASA ≥ 3 or BISAP ≥ 3 (Table 1).

**Table 1:** Distribution of patients as per sex, BISAP score, and ASA PS score Statistical test: Pearson Chi-Square (χ²) BISAP: Bedside Index of Severity in Acute Pancreatitis; ASA: American Society of Anesthesiologists; PS: physical status

Parameters	Group 1 (Preoperative criteria met) (n=83), n (%)	Group 2 (Preoperative criteria not met) (n=116), n (%)	p-value	χ2
Female sex	56 (67.5)	66 (56.9)	0.131	2.280
BISAP score ≥3	6 (7.2)	7 (6.0)	0.737	0.113
ASA score ≥3	21 (25.3)	33 (28.4)	0.802	0.319

Perioperative characteristics

The median time to surgery was four days (interquartile range (IQR) 3-6), with no significant difference between the two groups. There was also no difference in length of stay between the groups, with a median hospital stay of six days (IQR 4-8). The mean operative time was 79 minutes for patients meeting the preoperative criteria, and 83.4 minutes for those who did not meet criteria.

Postoperatively, there was no statistical difference in Clavien-Dindo complications (Table 2), including surgical site infection (2.4% vs 4.3%, p=0.46), haematoma (2.4% vs 2.6%, p=0.94), bile leak (0% vs 1.7%, p=0.14), and intra-abdominal collection (1.2% vs 3.4%, p=0.30). There were also similar rates of requirements for further intervention (10.8% vs 10.3%, p=0.93), including endoscopic retrograde cholangiopancreatography (ERCP) (7.2% vs 6.0%, p=0.73) and interventional radiology (1.2% vs 0%, p=0.19). There was no postoperative venous thromboembolism or mortality reported in this study cohort.

**Table 2:** Postoperative outcomes based on preoperative criteria Statistical test: Pearson Chi-Square (χ²) DVT: deep vein thrombosis; PE: pulmonary embolism; ERCP: endoscopic retrograde cholangiopancreatography

Parameters	Group 1 (Preoperative criteria met) (n=83), n (%)	Group 2 (Preoperative criteria not met) (n=116), n (%)	p-value	χ2
Surgical site infection	2 (2.4)	5 (4.3)	0.464	2.262
Haematoma	2 (2.4)	3 (2.6)	0.937	1.886
Bile leak	0 (0)	2 (1.7)	0.139	3.872
Intra-abdominal collection	1 (1.2)	4 (3.4)	0.297	2.179
DVT/PE	0 (0)	0 (0)	-	-
ERCP	6 (7.2)	7 (6.0)	0.737	0.113
Radiology	1 (1.2)	0 (0)	0.185	1.405
Further intervention (including ERCP)	9 (10.8)	12 (10.3)	0.927	0.008

## Discussion

There is strong support in the literature for index admission laparoscopic cholecystectomies in cases of mild AGP. Several systematic reviews and meta-analyses [12-19] identified benefits including: reduced (or no increase in) risk of complications or mortality compared to DLC, reduced time to surgery, shorter overall hospital stay, and fewer biliary events/complications (e.g., cholecystitis, cholangitis, biliary colic, recurrent gallstone pancreatitis). Isbell et al. also found that ELC had an 80% likelihood of reducing 90-day healthcare system costs [20]. In addition, a randomised controlled trial (RCT) by Davoodabadi et al. noted that there was no significant difference in operative difficulty in ELC vs DLC [21]. Other reviews have suggested that further high-quality RCTs are still required to investigate outcomes of ELC, particularly in cases of severe AGP [22].

There are discrepancies in the literature regarding what time frame constitutes an ‘early’ cholecystectomy for AGP, ranging from within 48-72 hours to one to two weeks. Aboulian et al. conducted a study in which participants were randomised to ELC within 48 hours regardless of their clinical condition (e.g. pain, biochemical markers), compared with a control group in which they aimed for an improved clinical condition prior to ELC [23]. They found that the intervention group had a shorter hospital stay, with no change in perioperative complication rate. Mueck et al. also conducted an RCT with similar study groups and found that ELC within 24 hours of admission, performed regardless of pain or laboratory markers, led to shorter length of stay as well as reduced rates of further intervention (including ERCP) [24]. However, they also noted a possible increase in Clavien-Dindo grade I-III complications in the intervention group. Nevertheless, ELC is still generally considered best practice, and in our study, the median time to index admission ELC was four days. 

The inclusion criteria of the PONCHO trial [8] were used to form the preoperative criteria used in this study for our sub-group analysis, as they were also used in clinical practice in certain circumstances to help guide decision-making for surgery. At our institution, the consensus among consultant surgeons is that the preferred timing for surgery is influenced by some or all of these three criteria, although there is some variation between individual surgeons. However, it is worth noting that the majority of participants in our study did not meet all three of the PONCHO trial inclusion criteria before proceeding to surgery. Of the three, CRP level seemed to influence the majority of cases, with only 21.6% of all study participants undergoing surgery with a CRP level >100 mg/L, which is also only 37% of the study group that did not meet at least one of the three preoperative criteria.

Our study did not demonstrate any significant differences between the two study groups in our primary outcome of postoperative Clavien-Dindo complications. In particular, there were similar rates of surgical site infection, haematoma, bile leak, and intra-abdominal collections. The main reasons for unplanned returns to theatre postoperatively were for haematomas or intra-abdominal collections, whilst the rates of ERCP requirement were also similar between the two groups.

The similar complication rates between our two study groups suggest that there is no significant difference in patient outcome based on the preoperative criteria from the PONCHO trial [8]. Therefore, this raises the question of whether all of these preoperative factors need to be met prior to undergoing cholecystectomy. If patients are suitable to undergo ELC (for example, still requiring opioid analgesia, not yet being on a solid diet, or with serum CRP level >100mg/L prior to surgery), while maintaining comparable outcomes and complication rates, this may allow earlier identification of patients ready for ELC. This would likely lead to reduced time to surgery, shorter lengths of hospital stay, and, therefore, potentially reduced individual healthcare costs.

Limitations

Our study has several limitations. The retrospective nature of the study means that we are reliant on the accuracy of previous documentation from the electronic medical records. In addition, the relatively small sample size also suggests that our results should be interpreted with caution, and they may not be generalisable to the wider population. The study is also relatively underpowered with a heterogeneous population, and further higher-powered research would be required to identify significant differences between study groups. 

The decision about timing of ELC was still at the discretion of each individual operating surgeon, and there was a lack of standardised criteria used to determine patient readiness for surgery. This resulted in variability in the time between patients meeting the preoperative criteria and the timing of their surgery. Furthermore, the retrospective cohort study design means that factors outside of the preoperative study criteria were not controlled, which could lead to confounding. In addition, the follow-up period was 30 days postoperatively, which could mean that more delayed complications were not included. 

## Conclusions

This study highlights the relatively low complication rates of ELC for AGP, as well as some of the difficulty in determining the appropriate time to proceed to ELC during the index admission. While patient factors (e.g., diet tolerance and pain level) and biochemical markers (e.g., serum CRP level) have been considered as indicators of readiness for surgery, using these has not necessarily demonstrated significant differences in patient outcomes or complication rates. It should therefore be considered whether ELC can be safely performed without waiting for all of these preoperative factors to be met, as this would likely lead to a reduced hospital stay and, therefore, potentially reduced individual healthcare costs. We suggest that ELC is still considered best and safe practice for AGP, and further higher-powered, prospective studies are required to investigate the optimal timing of cholecystectomy within the index admission, as well as its impact on patient outcomes and healthcare costs. 
